# Biological Aspects of mTOR in Leukemia

**DOI:** 10.3390/ijms19082396

**Published:** 2018-08-14

**Authors:** Simone Mirabilii, Maria Rosaria Ricciardi, Monica Piedimonte, Valentina Gianfelici, Maria Paola Bianchi, Agostino Tafuri

**Affiliations:** 1 Laboratory of Cell Kinetics and Applied Proteomics, Faculty of Medicine and Psychology, Department of Clinic and Molecular Medicine, Sapienza University of Rome, via Rovigo 1, 00161 Rome, Italy; simone.mirabilii@uniroma1.it (S.M.); mariarosaria.ricciardi@uniroma1.it (M.R.R.); monica.piedimonte@uniroma1.it (M.P.); gianfelici@bce.uniroma1.it (V.G.); mariapaolabianchi82@gmail.com (M.P.B.); 2 Hematology, “Sant’Andrea” University Hospital, Sapienza University of Rome, via di Grottarossa 1035, 00189 Rome, Italy

**Keywords:** leukemia, cell signaling, metabolism, apoptosis, miRNA, mTOR inhibitors

## Abstract

The mammalian target of rapamycin (mTOR) is a central processor of intra- and extracellular signals, regulating many fundamental cellular processes such as metabolism, growth, proliferation, and survival. Strong evidences have indicated that mTOR dysregulation is deeply implicated in leukemogenesis. This has led to growing interest in the development of modulators of its activity for leukemia treatment. This review intends to provide an outline of the principal biological and molecular functions of mTOR. We summarize the current understanding of how mTOR interacts with microRNAs, with components of cell metabolism, and with controllers of apoptotic machinery. Lastly, from a clinical/translational perspective, we recapitulate the therapeutic results in leukemia, obtained by using mTOR inhibitors as single agents and in combination with other compounds.

## 1. mTOR Structure and Function

**Figure 1:**
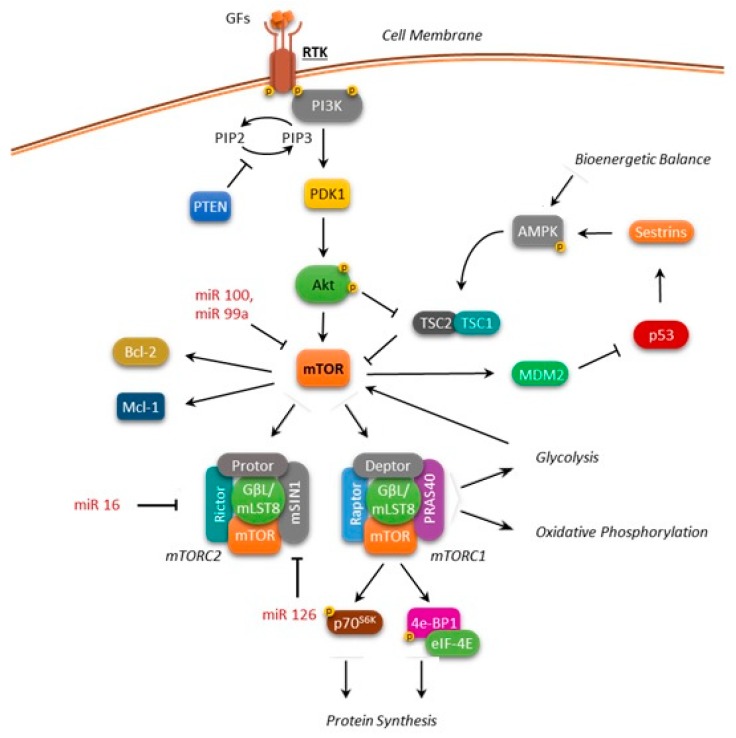
Overview of the mTOR regulation network. Arrows: positive interaction; T-bars: inhibition.

mTOR (also known as the mechanistic target of rapamycin) is a 289-kDa serine/threonine kinase belonging to the phosphatidylinositol kinase-related kinase (PIKK) family. Its COOH-terminal catalytic domain shows a very high homology to the phosphoinositide 3-kinase (PI3K) [1]. mTOR is structurally associated with other proteins forming two functionally distinct complexes, mTOR complex 1 (mTORC1) and mTOR complex 2 (mTORC2), characterized by a different response to rapamycin and its derivatives (rapalogs) [2,3]. mTORC1 includes mTOR, the regulatory-associated protein of mTOR (Raptor), the mammalian lethal with SEC13 protein 8 (mLST8), which stabilizes the kinase domain, and also the following inhibitory components: DEP domain-containing mTOR-interacting protein (Deptor), PRAS40 (proline-rich Akt substrate of 40 kDa), and FKBP38 (FK506-binding protein 38). mTORC1 is sensitive to rapamycin and its derivatives (Figure 1).

Similarly to mTORC1, mTORC2 also includes mTOR, mLST8, and Deptor, but contains the protein Rictor (Rapamycin-insensitive companion of mTOR) as a component, rather than Raptor. mTORC2 also associates with the mammalian stress-activated protein kinase interacting protein (mSIN1) and protein observed with Rictor-1 (Protor-1) (Figure 1).

Although rapamycin does not bind or directly inhibit mTORC2, it has been shown that prolonged treatment with rapamycin and its derivatives is able to abrogate mTORC2 signaling, probably due to the inability of mTOR linked to rapamycin to incorporate into new mTORC2 complexes [4,5]. mTORC1 and mTORC2 govern multiple cellular functions.

By integrating signals from the external environment with information on the metabolic status of the cell, mTORC1 controls the anabolic processes to promote protein synthesis and cell growth and inhibits autophagy [6]. Signal integration occurs at the level of the TSC1-TSC2 (tuberous sclerosis complex1-2) complex, the main inhibitor that is upstream of mTORC1. This complex acts as a molecular switch: during stress conditions, it suppresses mTOR activity thus limiting cell growth while it releases its inhibition under favorable conditions. TSC1 stabilizes TSC2, preventing its ubiquitin-mediated degradation.

Activated mTORC1 phosphorylates "protein synthesis machine” components that include p70S6K (protein S6 kinase beta-1) and 4E-BP1 [eukaryotic translation initiation factor 4E (eIF4E)-binding protein 1]. Following activation, p70S6K enhances messenger RNA (mRNA) translation by phosphorylating the 40S ribosomal protein, S6. Conversely, phosphorylation of 4E-BP1 at multiple sites promotes its dissociation from eIF4E and allows the initiation of cap-dependent translation [7].

The mechanisms related to the regulation of mTORC2 activity remain poorly understood. It has been documented that mTORC2 controls the cell proliferation and survival, and the organization of the actin cytoskeleton [3,8]. In particular, it has been reported that one of the main functions of TORC2 is the rapamycin-insensitive cell cycle-dependent regulation of actin polymerization through the activation of Rho GTPase [9]. Moreover, the reported mTORC2-ribosome association suggests its role in protein synthesis [10] and in driving the oncogenic PI3K signaling in cancer [8]. In fact, compelling evidence has shown that aberrant activation of mTOR is associated with the development and progression of several types of cancers [11]. Recently, mTORC2 has emerged as an additional regulator of cellular and tumor metabolism, prompting further investigation.

## 2. mTOR Deregulation in Leukemias

There is increasing evidence that deregulation of the PI3K/Akt/mTORC1 signaling contributes to leukemogenesis. Increased mTORC1 and mTORC2 activity has been reported to play a critical role in leukemia initiation, propagation, and relapse [12,13,14,15,16,17]. Particularly, mTOR constitutive activation is frequently found in leukemia patients, contributing to chemoresistance, disease progression, and unfavorable outcomes.

In the view of mTOR functioning as a point of convergence between a nutrient-sensing pathway (via-mTORC1) and as a regulator of Akt itself (via-mTORC2), the definition of the role of mTOR in controlling cellular metabolism and energy homoeostasis in normal and cancer cells plays a fundamental role in developing effective therapies for leukemia treatment (Figure 1). Over the years, several small molecules that target the PI3K/Akt/mTOR signaling pathway have been investigated, showing potential therapeutic efficacy in hematologic malignancies, alone or in combination with chemotherapeutic drugs.

The efficacy of mTOR inhibition in the treatment of various types of cancer is still being evaluated, and there are many possibilities that have yet to be explored in identifying areas where rapamycin might prove to be an effective treatment for cancer.

## 3. mTOR Involvement in Leukemia Metabolism

From a metabolic perspective, mTOR has been traditionally considered as a central regulator that is involved in the promotion of anabolic processes. Signals from bioenergetics status, oxygen levels, DNA damage, and amino acids availability converge on mTOR, unleashing a series of metabolic responses [18]. In fact, it has been shown that mTOR, when incorporated in mTORC1, promotes the synthesis of proteins, lipids, and nucleotides, as well as the adoption of a glycolytic phenotype, and an increase of carbon flux in the pentose phosphate pathway [18,19]. Indeed, research efforts in the leukemia setting have been mainly focused on the glycolytic aspect, in an attempt to exploit this feature as a target for therapeutic intervention. Many studies are focused on the connection between mTOR and glycolysis, mainly using 2-deoxy-d-glucose (2DG) for the inhibition of the glycolytic process. 2DG is a long-known glucose analog, which cannot be metabolized by cells, thus competing with glucose and accumulating in the cytosol [20]. Adenosine monophosphate (AMP)-activated protein kinase (AMPK) acts as a key sensor of cellular energy status coordinating multiple metabolic pathways in order to maintain the balance between ATP production and consumption [21]. Once activated by metabolic stresses, AMPK promotes ATP production switching on catabolic pathways while inhibiting macromolecules biosynthesis. Moreover, AMPK affects cell growth and proliferation by inhibiting mTOR and stabilizing p53 and p27 [21,22]. Pradelli, et al. observed that the blockage of glycolysis, either through 2DG exposure or through glucose deprivation, induces AMPK-mediated inhibition of mTOR, with a subsequent reduction of myeloid cell leukemia-1 (Mcl-1) and a sensitization toward the action of death receptor ligands on Jurkat acute lymphoblastic leukemia (ALL) and U937 acute myeloid leukemia (AML) cell lines [23]. This finding has been confirmed by Coloff, et al. on lymphoid cells, this time assessing the synergy between glycolysis inhibition and exposure to the Bcl-2 (B-cell lymphoma 2) inhibitor ABT-737 [24]. Similarly, Liu, et al. observed mTOR inactivation following 2DG exposure on myeloid cell lines that were previously treated with aurora kinase inhibitors [25]. In addition, Rosilio, et al. corroborated the observation that AMPK activation inhibits mTOR in T-ALL cells via the use of metformin, phenformin, and AICAR (5-Aminoimidazole-4-carboxamide ribonucleotide) [26]. Conversely, Estañ, et al. reported an opposite mechanism, where 2DG action in acute leukemia cell lines provokes AMPK inhibition and subsequent mTOR activation, along with a reduction in the intracellular ATP pool [27]. Interestingly, they observed a difference in the sensitization of leukemia cells when they were treated with arsenic trioxide and either 2DG or glucose deprivation, the latter being weaker, suggesting that 2DG may act through additional undetermined mechanisms [27]. However, despite the differences in the reported mechanism of action, all authors agree that mTOR action is paramount in controlling glycolysis in leukemia cells, allowing the conclusion that the block of this metabolic process is an effective therapeutic strategy, which confers sensitization to various chemotherapeutic agents [23,24,25,26,27]. Moreover, the action of agents targeting mTOR, such as rapamycin, appears to be enhanced when combined with glycolytic inhibitors [28,29].

Since glycolysis is the only reliable source of ATP on the fluctuating oxygen pressure condition that characterizes bone marrow [30], it is not unexpected that mTOR interacts with HIF1α (hypoxia-inducible factor 1-alpha), the master regulator of hypoxic response. Indeed, Konopleva’s group demonstrated that hypoxia conditions activate the Akt/mTOR pathway, while exposure to everolimus—one of the first mTOR inhibitors that were approved by the FDA for clinical use in the treatment of patients with cancer [31]—deactivates HIF1α, reverting the glycolytic phenotype of the ALL cell line [32]. Interestingly, Konopleva, et al. also reported that the Akt/mTOR pathway is strongly activated by co-culturing leukemia cells with mesenchymal stem cells in hypoxic conditions [32]; this observation is coherent with the idea that confers to mTOR the role of coordinating signals from microenvironment and subsequently adapting metabolism to these conditions. Accordingly, Brown, et al. confirmed the activation of mTOR, on AML cell lines and primary cells, caused by stromal co-culture, which gives rise to an upregulation of glycolysis; the authors identified the triggering signal in the chemokine CXCL12 (C-X-C motif chemokine 12), through a CXCR4 (C-X-C chemokine receptor type 4 )/mTOR axis [33].

A recent work by Feng and Wu identified the enzyme phosphofructokinase-2/fructose-2,6-bisphosphatase 3 (PFKFB3) among the downstream targets of mTOR [34]; it is directly involved in the glycolytic process, mediating the conversion of fructose 6-phosphate in fructose 2,6-bisphosphate (and vice versa). The latter in turn regulates phosphofructokinase-1, which controls the critical step of glycolysis, the conversion of fructose 6-phosphate in fructose 1,6-bisphosphate [35]. This interaction could explain the mechanism by which mTOR controls glycolysis.

Alternatively, it has been reported that mTOR has a deep impact on mitochondrial respiration, activating oxidative phosphorylation through a mechanism involving its association with Raptor in Jurkat cell lines, whereas exposure to rapamycin disrupted the association and caused a decrease in cellular oxygen consumption [36]. The exposure to agents that impaired mitochondrial membrane potential inactivated mTOR, thus suggesting the presence of a feedback-controlling mechanism [37]. Ramanathan and Schreiber confirmed this finding, showing a downregulation of mitochondrial metabolism coupled with an upregulation of glycolysis when the Jurkat cell line is exposed to rapamycin [38]. These two reports appear to be in contrast with the previously mentioned mTOR activation of glycolysis. However, the use of the same cell model may limit these studies, as these results have not been expanded to other cell lines and/or primary samples. On the other hand, it may be possible that other layers of regulation exist, thus prompting further studies to better clarify the different roles of mTOR in the choice between glycolytic and mitochondrial metabolism.

The mTOR control over the metabolic phenotype has been associated with the resistance of leukemia cells to various agents. Beesley, et al. characterized the transcriptional profile of a panel that was composed of glucocorticoid-resistant T-ALL cell lines, showing that these are characterized by exceptionally high expression of genes related to both glycolysis and oxidative phosphorylation [39]. Exposure to rapamycin restored cell sensitiveness to dexamethasone, indirectly linking metabolism upregulation and mTOR activity [39]. More compelling evidence has been presented by Sharma, et al. who detected mTOR hyperactivation in B-cell leukemia and lymphoma cell lines, and in primary cells that were resistant to fludarabine, which is associated with higher rates of glycolysis and oxidative phosphorylation [40]. The role of mTOR has been verified in the acquisition of this metabolic phenotype, since everolimus was able to revert this higher metabolism [40]. Similar data were generated by our group: exposure to NVP-BKM120, a pan class PI3K inhibitor, on AML cells brought to mTOR deactivation, followed by a decrease in both glycolysis and oxidative phosphorylation [41]. Interestingly, Nepstad, et al. characterized metabolic differences between primary AML cells sensitive and resistant to PI3K/Akt/mTOR inhibitors, including rapamycin, tracing them among glutamine and lipid metabolism [42]. Taken together, these results seem to suggest that (i) leukemic cells display a high degree of heterogeneity in their metabolism; (ii) those which turn out to be resistant to therapeutic agents are generally characterized by a highly active metabolism; (iii) mTOR is the master regulator of leukemic cell metabolism, but we are only beginning to understand the mechanisms by which it operates in the leukemia cell setting.

## 4. mTOR and Apoptosis Regulation in Leukemias

Among the many functions previously listed, mTOR indirectly inhibits apoptosis [43,44] through a mechanism that depends on the cellular context and on the control of target molecules such as p53, Bcl-2, BAD (Bcl-2-associated death promoter), p21, p27, and c-myc [45].

### 4.1. mTOR and p53

**Figure 2:**
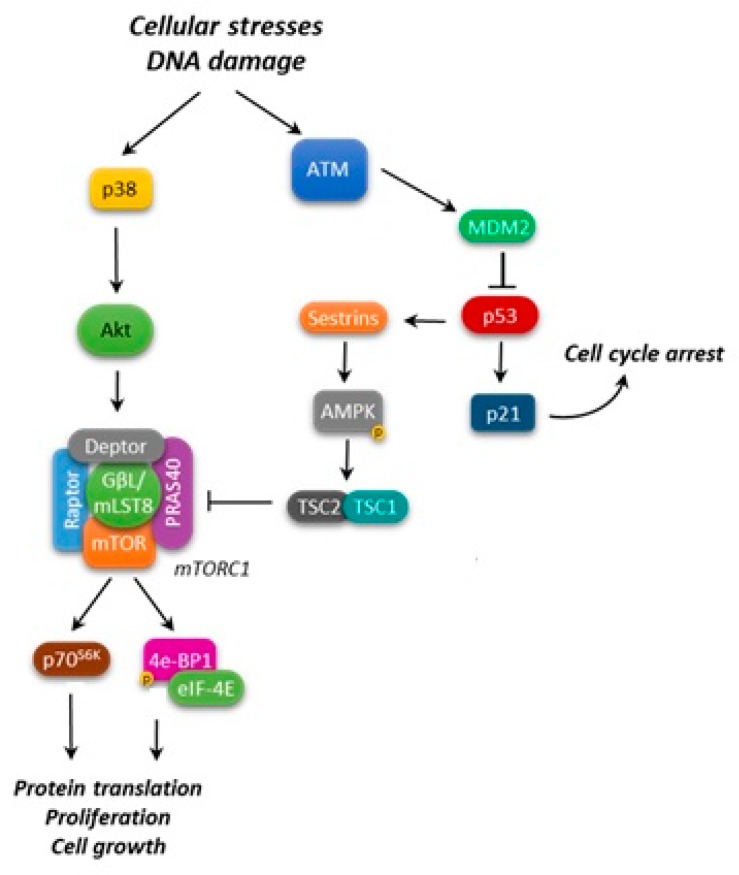
p53 and mTORC1 interaction. Arrows: positive interaction; T-bars: inhibition.

Recent studies suggest a multifaceted integration of p53 and mTORC1 pathways to drive successful cell growth and proliferation, while in the meantime preserving genome integrity (Figure 2) [46].

As a key point in such an intricate cooperation, p53 acts in balancing mTORC1 pro-growth activity. In response to cellular stresses and DNA damage, p53 inhibits mTORC1 activity through the upregulation of mTORC negative regulators, such as the activation of AMPK [47], the induction of PTEN (phosphatase and tensin homolog) and TSC2 transcription [47,48], and the decrease of S6K1 (ribosomal protein S6 kinase beta-1) activity or the dephosphorylation of 4E-BP1 [49]. In particular, glucose deprivation in normal cells causes AMPK to inhibit mTORC1 and stabilize p53, stalling cell growth and division [50].

Together, these observations highlighted that p53 inhibits mTOR by regulating a pathway that is used to detect nutrient/energy deprivation such as AMPK, and subsequently the TSC1/TSC2 complex [47].

More recently, it has been clarified that the products of two p53 target genes, Sestrin1 and Sestrin2, negatively regulate mTOR signaling by activating AMPK, which in turn phosphorylates TSC2 [51].

Nonetheless, it has been reported that lymphoma lesions, such as mutant EZH2 (enhancer of zeste homolog 2) in follicular lymphoma (FL), can override the p53-mediated induction of Sestrin1. Specifically, using a chimeric mouse model of lymphoma, Oricchio, et al. [52] demonstrated that Sestrin1 is epigenetically silenced by the lymphoma-specific mutant EZH2Y641X, resulting in mTORC1 activation. The reported EZH2-mediated epigenetic down-regulation of Sestrin1 increases the dependency of lymphomas on mTORC1 and induces sensitivity to mTORC1 inhibitors in EZH2 mutant lymphomas. On the other hand, it has also been reported that in cells that are exposed to genotoxic agents p38α/mTORC1/S6K1 down-regulate MDM2 (mouse double minute 2 homolog) levels, thereby inhibiting p53 ubiquitination and increasing p53 function [53]. This additional genotoxic stress-responsive pathway can contribute to regulate the p53-mediated cell response to DNA damage by sensing the cells' nutrient and energy status.

In a chronic myeloid leukemia (CML) model using the K562 cell line, which is resistant to imatinib, it was found that an increase of phospho-p70S6K and a decrease of phospho-p53Ser15, Bax (Bcl-2-associated X protein), and active caspase-3 compared to the wild-type K562 cell line occurred [54]. The authors also reported that dasatinib was able to induce p-p53Ser15 and active caspase-3 expression, thus promoting apoptosis by the downregulation of AktSer473 and the inhibition of mTOR/p70S6KThr389 activity [54].

Therefore, p53 and mTOR pathway activity could be integrated and combined to inhibit cell growth in response to DNA damage. Deciphering p53 and mTOR crosstalk could be the key to understanding their role in cancer suppression, and the potential clinical usefulness of mTORC1 inhibitors and modulators of the MDM2/p53 module.

Aberrant activation of the PI3K/Akt/mTOR pathway, as well as inactivation of wild-type p53 by MDM2 overexpression, are frequently observed in AML [55,56,57]. Kojima, et al. demonstrated that the simultaneous inhibition of PI3K/Akt/mTOR axis and of MDM2 induced the dephosphorylation of 4E-BP1, a decrease in MDM2, p21, Noxa, and Bcl-2 expression, and the conformational change of Bax, thus affecting mitochondrial stability and enhancing p53-mediated mitochondrial apoptosis in p53 wild-type AML [58]. Moreover, the use of a dual PI3K/mTOR inhibitor is more effective in blocking two separate inputs that promote mTOR activation: growth factors that stimulate mTOR through the class 1 PI3K, and the nutrients that activate mTOR through a class 3 PI3K.

Independent from a direct interaction between p53 and mTOR in cell death control, the effectiveness of their simultaneous modulation has been highlighted in leukemia. For example, Guo, et al. [59] reported that the reactivation of p53 by Nutlin-3, and Akt/mTOR inhibition by tanshinone IIA, exhibit a synergetic anti-leukemia effect with imatinib in Philadelphia positive (Ph +) ALL.

### 4.2. mTOR and Bcl-2 Family

**Figure 3:**
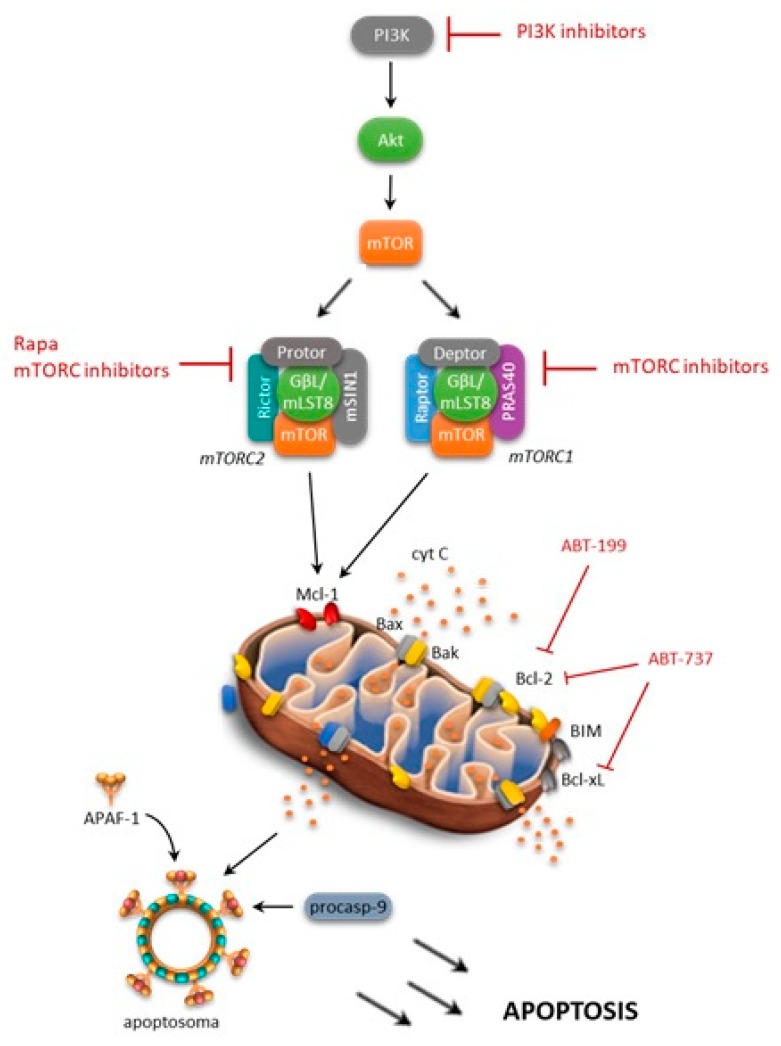
mTOR and Bcl-2 family member interactions. Arrows: positive interaction; T-bars: inhibition.

Several research groups have shown that a high level mTOR expression is able to control apoptosis by modulating several molecules, including Bcl-2 family members, and thus promoting tumor cell survival (Figure 3) [44,45,60]. The activated PI3K/Akt/mTOR pathway has been shown to decrease the BH3 (Bcl-2 homology domain) mimetic effectiveness in cancer cells by upregulating anti-apoptotic Bcl-2 family members, such as Mcl-1 [61]. Consistently, rapamycin, an inhibitor of mTOR, has been shown to induce a decrease in Mcl-1 expression, and this mechanism is able to overcome drug resistance [62]. Moreover, it was reported that NVP-BEZ235, a PI3K/mTOR dual inhibitor, decreases Mcl-1 expression and sensitizes ovarian carcinoma cells to Bcl-xL (B-cell lymphoma-extra large)-targeting strategies. Subsequently, simultaneous blockade of PI3K/mTOR and the Bcl-2 pathway was shown to have promising antileukemic activity in leukemia cell lines, primary samples, and xenograft models. Rahmani and colleagues [61] have demonstrated that co-exposure to the dual PI3K/mTOR inhibitor NVP-BEZ235 and the Bcl-2/Bcl-xL inhibitor ABT-737 strongly potentiated the cytotoxicity of single agents in AML. They found that antileukemic synergism involved multiple mechanisms, including Mcl-1 downregulation, the release of Bim (Bcl-2-like protein 11) from Bcl-2/Bcl-xL as well as Bak (Bcl-2 homologous antagonist/killer) and Bax from Mcl-1/Bcl-2/Bcl-xL, and GSK3α/β (glycogen synthase kinase 3 alpha/beta), culminating in a strong apoptosis induction [61].

In a similar fashion, Spender, et al. suggested that the combined use of BH3 mimetics with mTORC1/2 inhibitors could be a novel and effective therapeutic approach for the management of Burkitt's lymphoma [63]. Authors demonstrated that exposure of Burkitt's lymphoma cells to dual PI3K/mTOR inhibitors, such as PI 103, was associated with an increase in Bim/Mcl-1 expression ratios and the loss of c-myc expression [63]. Remarkably, they also observed that dual PI3K/mTOR inhibitors or mTOR active site inhibitors were effective in overcoming resistance to ABT-737, thus indicating that inhibition of cap-dependent translation regulated by 4E-BP1/eIF4E represents a key point to overcome BH3 mimetics resistance.

Our group has previously explored a combined in vitro targeted approach to overcome ABT-737-acquired resistance in ALL with the simultaneous inhibition of the mTOR pathway by CCI-779 [64]. We observed that simultaneous inhibition strongly enhanced the cytotoxicity of single agents sensitizing ALL cells to apoptosis, and reverting the acquired ABT-737 resistance of cell models and primary samples, while sparing normal progenitors. Analysis of signaling modulations in newly sensitized ABT-737 resistant cells reveals that ABT-737 plus CCI-779 combination reduces 4E-BP1 phosphorylation and Mcl-1 expression. Since the involvement of proteasomal degradation was excluded, the inhibition of 4E-BP1-dependent protein translation may be responsible for the synergistic interaction between ABT-737 and CCI-779. However, in some ALL models, Mcl-1 may be not the unique determinant of ABT-737 resistance, since knockdown of protein expression by RNA interference does not result in significant changes of cytotoxicity [64].

A similar approach was used by Rahmani, et al. [65], which demonstrated the cooperation between the selective Bcl-2 inhibitor and PI3K inhibition (venetoclax/GDC-0980) in inducing Bax-dependent apoptosis, which exhibiting strong anti-AML activity both in vitro and in vivo, as well as against multiple forms of venetoclax resistance.

More recently, by the high-throughput profiling of signaling networks, Andreeff’s group demonstrated that concomitant treatment with temsirolimus plus ABT-737 or the MDM2 inhibitor Nutlin-3a, is effective for eliminating microenvironmental resistance in AML, and it facilitated leukemic cell death [66].

Finally, it is interesting to mention the reported unsuspected role of mTOR as an apoptotic inducer. Indeed, Calastretti, et al. [67,68] stated that in human FL B-cell lines, characterized by high concentration of Bcl-2 protein, rapamycin increases the cellular concentration of p27kip1 and Bcl-2, leading to cell arrest in the G1 phase, and to the activation of an anti-apoptotic program. Moreover, prior treatment with rapamycin completely inhibited the taxol-induced apoptosis of human B-cell lines by phosphorylation/inactivation of Bcl-2.

This observation highlights the role of mTOR for a coordinated regulation of the cell cycle and apoptosis in normal and leukemia cells.

## 5. mTOR and MicroRNA Regulation in Leukemias

MicroRNAs (miRNAs) are ubiquitous regulators of biological processes that are involved in cellular differentiation, development, stress response, apoptosis, and cell growth. A number of studies have revealed differential expression of known miRNAs in different hematopoietic cell types, demonstrating that miRNAs play an important role in the decision by hematopoietic stem cells (HSC) and progenitor cells to self-renew or to differentiate into a specific cell type [69,70,71].

Most of these miRNAs have been reported to regulate the expression of key genes in the PI3K/Akt/mTOR pathway, and they are involved in the development and pathogenesis of hematological malignancies.

Among the miRNAs that directly target mTOR, miR-126 plays important functions in HSC, preserving quiescence and increasing self-renewal [70,72]. It is both highly expressed and functionally active within the murine and human HSC compartments, with progressive downregulation during the early steps of hematopoietic commitment. Lechman, et al. [70,72] reported interesting results that showed that miR-126 is involved in mTOR regulation by targeting mSIN1, together with several other members of the PI3K/Akt/mTOR axis. In particular, mSIN1 repression impairs the mTORC2 complex formation and full activation of Akt. They also showed that myeloid leukemia stem cells (LSC) express high endogenous levels of miR-126, compared with more differentiated AML populations [72]. Upregulation of miR-126 plays a critical role in promoting chemotherapy resistance of LSC.

Furthermore, mTOR and Rictor are direct targets of let-7 and miR-16. Marcais, et al. [73] showed that these miRNAs regulate the expression of mTOR components in CD4 T-cells and contribute to discriminate between T-cell activation and anergy.

MiR-100 and miR-99a are involved in mTOR regulation in ALL and contributed to the poor response to chemotherapy. Li, et al. [74] reported a downregulation of miR-100 and miR-99a in the cases of ALL, especially in T-ALL, and in ALL harboring mixed lineage leukemia (MLL) gene rearrangements and BCR-ABL1 fusion transcripts. Cases with lower levels of expression displayed shorter survival and a poorer outcome when compared to cases with higher expression. These miRNAs contribute to leukemia developments and progression by directly targeting the insulin-like growth factor 1 receptor (IGF1R) and mTOR. In fact, the protein levels of IGF1R and mTOR were reduced in Jurkat cells that were transfected with miR-100/99a mimics, while they were increased in cells that were transfected with the miR-100/99a inhibitors (antisense). Functional studies also showed that upregulation of mTOR induced the activation of Mcl-1 with consequent improvement of cell proliferation and inhibition of apoptosis.

Besides miRNAs which directly target mTOR components, several other miRNAs have been reported to targets that are upstream (mir-22, miR-26, miR-150, miR-193, miR-223, miR-3151) [75,76,77,78,79] or downstream (miR-29, miR-181) [80,81,82] of mTOR signaling, highlighting the importance of the PI3K/Akt/mTOR axis in leukemias.

## 6. mTOR Axis Inhibition in Leukemias

The role of the PI3K/Akt/mTOR pathway in leukemias has been widely studied, both for in vitro and in vivo models, in order to explore the therapeutic perspective of its inhibition. In fact, the use of the dual PI3K/mTOR inhibitor, dual Akt/tyrosine-kinase receptor (RTK) inhibitor, Akt inhibitor, selective inhibitor of PI3K, mTOR inhibitor, and the dual PI3K/phosphoinositide-dependent protein kinase-1 (PDK1) inhibitor in chronic and acute leukemias seem to have remarkable therapeutic effect as compared to conventional treatments [83].

### 6.1. Rapamycin and Rapalogs

Literature data have reported on first- and second-generation mTOR inhibitors. Among the first generation inhibitors, rapamycin and its analogs, called rapalogs, are the most well studied drugs, and they are now clinically used as cancer treatments. Rapamycin (sirolimus) is a macrolide antibiotic that is produced by the microorganism *Streptomyces hygroscopicus*, which has been discovered in 1975 as a potent antifungal agent [84]. In following studies, it was also associated with immunosuppression [85], which subsequently led to its development as a clinically useful drug in consideration of its anticancer activity [86]. Similarly to the immunosuppressant FK506, rapamycin binds to the intracellular receptor FKBP12 (FK506-binding protein 12), and they both share similar chemical structures. Nevertheless, the two macrolides have different mechanisms of action in cells, since FK506 inhibits T cell proliferation, whereas rapamycin interferes with cytokine signaling. However, the exact mechanism of how these interactions lead to inhibition of mTOR pathway remains to be understood.

In consideration of some pharmacological limitations of rapamycin, such as poor water solubility and chemical stability, rapalogs, including temsirolimus (CCI-779), everolimus (RAD001), and ridaforolimus (AP23573) have been developed, with minor immunosuppressive action [87]. Unfortunately, several studies have demonstrated that first generation inhibitors display limited anticancer activity, and that is partially due to the fact that the inhibition of mTORC1 by these drugs might lead to Akt upregulation and outgrowth of more aggressive lesions. Targeted strategies by combining rapamycin plus an inhibitor of Akt or PI3K, or the use of agents that target both PI3K and mTOR, or both mTORC1 and mTORC2, were then developed to circumvent this loop [88].

### 6.2. Dual Inhibitors 

Selective dual mTORC1 and mTORC2 inhibitors are second-generation mTOR inhibitors. They are ATP-competitive inhibitors of mTOR that block the phosphorylation of all downstream targets of both mTORC complexes without inhibiting other kinases [89]. Among the second-generation mTOR inhibitors, AZD8055 is a compound that is able to prevent mTORC2-mediated Akt activation. This agent has been evaluated in a multicenter phase I study including patients with advanced solid tumors or lymphomas [90]. The most frequent adverse events (AEs) were elevated transaminases and fatigue, and not hypercholesterolemia or hypertriglyceridemia, as is often reported in patients who are treated with other mTOR inhibitors, including rapalogs. Another second-generation mTOR inhibitor is CC-223, which was evaluated in pre-treated patients with advanced solid tumors or multiple myeloma (MM) [91]. Common CC-223-related AEs were fatigue, nausea, diarrhea, and hyperglycemia. TAK-228, another dual mTORC1/mTORC2 kinase inhibitor, has been tested in patients with MM, non-Hodgkin lymphoma and Waldenström's macroglobulinemia (WM) [92]; reported toxicities included thrombocytopenia, fatigue, and neutropenia. Unfortunately, the best response achieved in a WM patient has been a partial remission (PR).

Dual PI3K/mTOR inhibitors are small molecules that are able to block the ATP binding sites of mTOR and PI3K, thus targeting all the three key enzymes, PI3K, Akt, and mTOR [93], although data from clinical trials are not entirely satisfying. In particular, NVP-BEZ235 has been tested in adult patients with relapse/refractory (R/R) acute leukemia, showing an anti-leukemic efficacy; this was more pronounced in an ALL setting, granting an encouraging overall response rate and a sustained molecular remission [94].

### 6.3. mTOR Inhibitors in AML 

The activation of PI3K/Akt/mTOR has been observed in up to 80% of AML cases and has been associated with a poor prognosis [95].

Given the heterogeneity of leukemias, it is important to remark that there is variability within leukemias, in both in vitro and in vivo sensitivities to PI3K/Akt/mTOR inhibition. In this respect, Reikvam, et al. [96] investigated the functional effects on primary AML cells of two mTOR inhibitors (rapamycin, temsirolimus) and two PI3K inhibitors (GDC-0941, 3-methyladenine). They demonstrated that the antileukemic effect of PI3K/Akt/mTOR inhibition varies between patients. By unsupervised hierarchical clustering analysis, two main clusters were identified in this study: one included patients that exhibited remarkable antiproliferative effects to all inhibitors tested; the other patients proved to be resistant. Notably, resistant patients were characterized by a higher expression of *CDC25B*, a gene encoding a phosphatase involved in cell cycle progression [96,97]. In a recent study on AML patients, the same group identified two clusters that were characterized by high and low constitutive PI3K/Akt/mTOR activation [98]. Complex phenotypic differences, including genetic and molecular processes, cellular communication, and interactions with the microenvironment are reported to be involved in differences in pathway activation [98].

With regard to the heterogeneity of leukemias, additional crucial points that are related to the efficacy of targeted therapies are the leukemia-microenvironment crosstalk and subsequently the stem cell niche, a specialized microenvironment that helps to maintain stem cell characteristics [99,100]. Common pathways are potentially shared across multiple leukemias and are involved in microenvironment/leukemia interactions. Reikvam and colleagues [101] demonstrated that PI3K and mTOR inhibitors exerted direct and indirect antileukemic activity through the inhibition of angioregulatory mediators released by both AML and stromal cells. These effects are however mediated through the common targets represented by the PI3K/Akt/mTOR pathway.

The efficacy of several selective PI3K/Akt/mTOR inhibitors has been investigated on both AML cell lines and AML primary cells using mTOR inhibitors as single agents or in association with chemotherapy (Table 1). Recher, et al. [102] used rapamycin in monotherapy for patients with R/R de novo or secondary AML, showing partial responses in four out of nine patients. Perl, et al. [103] used rapamycin with the chemotherapy regimen MEC (mitoxantrone, etoposide, cytarabine) in patients with R/R or untreated secondary AML, reaching 22% of clinical responses (complete remission (CR) or PR), showing low synergy between rapamycin and chemotherapy. Furthermore, everolimus has been evaluated in association with low doses of cytarabine (LDAC) in naïve elderly AML patients who were not eligible for intensive chemotherapy [104]. Amadori, et al. [105] have combined temsirolimus with low dose of clofarabine as a salvage therapy. Temsirolimus has also been used for maintenance therapy in patients, achieving CR with or without full hematological recovery after induction (CRi). Despite these promising findings, the combination of rapalogs and chemotherapy failed to display the expected synergistic cytotoxicities in clinical trials.

In acute promyelocytic leukemia (APL), PI3K/Akt signaling is constitutively activated, and cells that are exposed to all-trans retinoic acid (ATRA) seem to be very sensitive to class I PI3K, p110beta, or p110delta inhibitors, and to rapamycin [83,106]. Therefore, PI3K and mTOR inhibitors in association with induction treatment regimens may provide therapeutic benefits [106]. In addition, the PI3K/Akt/mTOR pathway contributes to ATRA-induced granulocytic differentiation (Table 1) [107].

### 6.4. mTOR Inhibitors in ALL

The hyperactivation of the PI3K/Akt/mTOR pathway was reported also in B-ALL, where encouraging results have been obtained with the inhibitors of PI3K/mTOR and MEK1/2 [108]. In the study of Messina, et al. [108], ALL cells with mutated *NRAS* or *KRAS* showed sensitivity to rapamycin and to the dual PI3K/mTOR inhibitor NVP-BEZ235. Thus, the authors pointed out PI3K/mTOR inhibitors, among others, as alternative therapeutic approaches in frail patients (Table 2). Other selective mTORC1 inhibitors inducing apoptosis in ALL are RAD001, Torin-2, and CCI-779. RAD001 shows also a potent synergic effect with the Akt allosteric inhibitor MK2206 [83]. Instead, in the Ph + ALL setting, it has been shown that BCR-ABL is able to activate the survival pathway PI3K/ Akt/mTOR [109]. The use of the mTOR inhibitor and in combination with imatinib has also been proven to have a synergic effect even in imatinib-resistant cell lines [109].

The PI3K/Akt/mTOR pathway is also constitutively active in numerous T-ALL patients and this affects the patient outcome, indicating it as a potential therapeutic target for T-ALL. T-ALL, which represents 15% of pediatric ALL and 25% of adult ALL, is an aggressive disease where relapses are not infrequent, despite the good response to chemotherapy. The very poor prognosis suggests the need for new therapeutic strategies. The negative PI3K/mTOR pathway regulator, PTEN, is frequently mutated in T-ALL, leading to hyperactivation of the pathway [110]. The combination of rapamycin with the chemotherapeutic agent dexamethasone shows a synergic effect in T-ALL cells [111]. In addition, numerous pathway inhibitors, such as GDC-0941 (a pan class I PI3K inhibitor), MK-2206 (an allosteric Akt inhibitor), RAD001 (an mTORC1 inhibitor) and the dual PI3K/PDK1 inhibitors NVP-BAG956 and NVP-BEZ235, show a potent cytotoxic effect in T-ALL cell lines, as well as in patient-derived cells [112].

The NOTCH pathway, altered in about 50% of T-ALL patients [110], triggers the upregulation of the PI3K/Akt pathway through the transcription factor HES1 (hairy and enhancer of split-1), which negatively regulates the expression of PTEN [113]. Mutations of PTEN confer resistance to treatment with GSIs (gamma-secretase inhibitors) that blocks the NOTCH1 (Notch homolog 1, translocation-associated) pathway [113]. This interplay between NOTCH1 and PTEN suggests the possible efficacy of a combined inhibition of PI3K/Akt and the NOTCH1 pathway in T-ALL.

### 6.5. mTOR Inhibitors in Other Leukemias

The PI3k/Akt/mTOR pathway is one of the multiple signaling pathways that are activated by BCR-ABL in CML cells, so drugs targeting key molecules such as PI3K, Akt and mTOR have been reported to exert beneficial effects in CML progenitor and stem cell populations (Table 1). These drugs show synergic activity with tyrosine kinase inhibitors (TKis). In particular, the dual PI3K/PDK1 inhibitor NVP-BEZ235 is able to sensitize CML stem cells and progenitors to nilotinib, enhancing its cytotoxicity in TKi-resistant BCR-ABL mutant cells [114]. Moreover, a combination of dasatinib with rapamycin or LY294002 decreases FOXO1/3 (forkhead box proteins O1 and O3) phosphorylation and drives the apoptosis of CML cells [115]. Resveratrol, a phytoalexin, and a natural phenol produced by several plants, acts downstream of BCR-ABL, and inhibits Akt activity [116]. Conversely, in accelerated phase/blastic phase (AP/BP) CML patients, increased ABCG2 (drug pump, ATP-binding cassette sub-family G member 2) expression was associated with the lack of PTEN protein and subsequent Akt activation [117]. This suggests that PI3K/Akt could be an alternative therapeutic target in CML, since ABCG2 seems to be regulated by PTEN through the PI3K/Akt pathway [117]. TKi can also abrogate the activation of PI3K/Akt/mTOR, and therefore in the TKi-resistant cells, simultaneous inhibition of PI3K and Akt/mTOR is recommended to obtain a potent pro-apoptotic effect in CML cells.

Concerning chronic lymphocytic leukemia (CLL), one of the major prognostic factors is the specific characteristic of the B-cell receptor (BCR), upstream of a signal transduction pathway that is essential for survival and proliferation, with a major role in the context of prognosis and positive selection of the precursor tumoral cell. In fact, antigenic stimulation and therefore the constitutive activation of BCR signaling plays a fundamental role in the pathogenesis of CLL [118].

BCR stimulation triggers the activation of numerous intracellular pathways that regulate normal B cells or leukemia. In fact, BCR acts on kinases, such as spleen tyrosin kinase (SYK) and SRC kinase LYN, which phosphorylate immune receptors belonging to the complementary proteins of the BCR, CD79a, and CD79b complexes. This phosphorylation causes the recruitment of adaptive proteins and other kinases, such as Bruton tyrosine kinase (BTK) or PI3K to a complex formed by the accessory proteins of BCR and then it determines the downstream stimulation of Akt/mTOR, NF-kB (nuclear factor kappa-light-chain-enhancer of activated B cells), and/or ERK (extracellular-signal-regulated kinase) [119].

CC-115, a novel dual mTOR kinase and a DNA-dependent protein kinase (DNA-PK) inhibitor, was evaluated [120]. CC-115 showed encouraging preclinical data on the ability to overcome resistance to chemotherapy or venetoclax, as well as to idelalisib, proving to be an attractive compound for further combination studies in clinical settings (Table 2) [120].

In conclusion, the use of rapalogs, or of second-generation mTOR inhibitors—developed with the aim to overcome the weaknesses of rapalogs—showed a limited impact, despite the expected benefits in clinical trials. This could be due to several mechanisms, such as an incomplete blockage of the pathway, or the existence of feedback loops. Thus, these observations should prompt further studies that address the clarification of the mechanisms of drug resistance, and the design of a more precise and personalized treatment.

## 7. Summary

Over the last decade, it has been widely demonstrated that the mTOR pathway is physiologically activated during various cellular processes and that it is deregulated in human diseases such as cancer. The critical role of mTOR in leukemia initiation, progression, and chemoresistance has also been proven by scientific and clinical studies evaluating its down-modulation. To date, several classes of pharmacological agents targeting the mTOR network have been developed. Each of them has their own pharmacokinetic properties, inhibitory activities, and toxicity profiles. Therefore, understanding which inhibitor is more clinically effective in each patient remains a constant challenge. The answer depends on the recognition of specific oncogenic addiction profile of leukemia cells, thus the need of new biomarkers for patient selection. The advances in the comprehension of the biological impact that mTOR has in the leukemia setting, described in this review, could contribute to the potential future development of effective mTOR-targeting based therapeutics.

## Figures and Tables

**Table 1:** mTOR inhibitors in myeloid leukemias.

Inhibitors	Disease	Response	Reference
Rapamycin monotherapy	R/R AML	PR	Recher, et al. [102]
Rapamycin + MEC	R/R or untreated secondary AML	CR/PR	Perl, et al. [103]
Everolimus + LDAC	Naïve elderly AML	CR/Cri/PR	Wei A.H., et al. [104]
Temsirolimus + LDClof	R/R AML	CR/Cri	Amadori, et al. [105]
ATRA + LY294002 and PD98059	APL	Increase granulocyte differentiation	Scholl S., et al. [106]
NVPBEZ235 + nilotinib	TKI-resistant BCR-ABL	Increase apoptosis	Airiau K., et al. [114]
Rapamycin + dasatinib	CML	Increase apoptosis	Pellicano F., et al. [115]
Resveratrol	CML	Inhibits Akt	Banerjee M. S., et al. [116]

**Table 2:** mTOR inhibitors in lymphoid leukemias.

Inhibitors	Disease	RESPONSE	Reference
CC-115	R/R CLL	PR	Thijssen, et al. [120]
Rapamycin + NVP-BEZ235	ALL	Increase apoptosis	Messina, et al. [108]
RAD001, Torin-2 and CCI-779	ALL	Increase apoptosis	Bertacchini, et al. [83]
Imatinib + mTOR inhibitor	Imatinib-resistant Ph + ALL	Increase apoptosis	Xing H., et al. [109]
